# A joint deep learning model to recover information and reduce artifacts in missing-wedge sinograms for electron tomography and beyond

**DOI:** 10.1038/s41598-019-49267-x

**Published:** 2019-09-05

**Authors:** Guanglei Ding, Yitong Liu, Rui Zhang, Huolin L. Xin

**Affiliations:** 1 Department of Physics and Astronomy, University of California, Irvine, CA 92697 United States; 2 School of Information and Communication Engineering, Beijing University of Posts and Telecommunications, Beijing, 100876 China

**Keywords:** Transmission electron microscopy, Imaging techniques

## Abstract

We present a joint model based on deep learning that is designed to inpaint the missing-wedge sinogram of electron tomography and reduce the residual artifacts in the reconstructed tomograms. Traditional methods, such as weighted back projection (WBP) and simultaneous algebraic reconstruction technique (SART), lack the ability to recover the unacquired project information as a result of the limited tilt range; consequently, the tomograms reconstructed using these methods are distorted and contaminated with the elongation, streaking, and ghost tail artifacts. To tackle this problem, we first design a sinogram filling model based on the use of Residual-in-Residual Dense Blocks in a Generative Adversarial Network (GAN). Then, we use a U-net structured Generative Adversarial Network to reduce the residual artifacts. We build a two-step model to perform information recovery and artifacts removal in their respective suitable domain. Compared with the traditional methods, our method offers superior Peak Signal to Noise Ratio (PSNR) and the Structural Similarity Index (SSIM) to WBP and SART; even with a missing wedge of 45°, our method offers reconstructed images that closely resemble the ground truth with nearly no artifacts. In addition, our model has the advantage of not needing inputs from human operators or setting hyperparameters such as iteration steps and relaxation coefficient used in TV-based methods, which highly relies on human experience and parameter fine turning.

## Introduction

**Figure 1:**
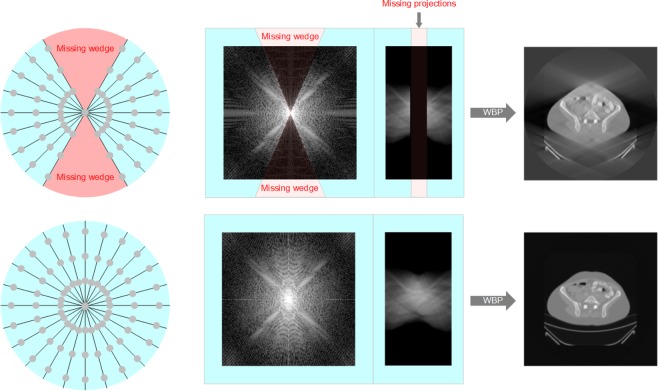
The missing-wedge problem in electron tomography.

The reconstruction of tomography images or tomograms has great significances for physical, materials, medical sciences because it offers capabilities to investigate the internal structures of a non-transparent object without having to dissect or disrupt it. Tomography is performed by taking a series of projection images of a three-dimensional (3D) object around a fixed tilt axis to form a sinogram. By inverse Radon transform the obtained sinogram, a tomogram, i.e. the cross-sectional images, showing the density and morphological structure inside an object can be reconstructed. However, in many practical applications, it is difficult or not possible to obtain a complete set of projection images with full rotations from −180° to +180°, due to limitations on hardware conditions, radiation dose, or the state of the object being imaged. In transmission electron microscopes (TEM), for example, the distance between the electromagnetic lenses is only a few millimeters. Given the TEM samples are typically 3 mm in size, the limited space imposes a physical limitation on the tilt range. Even when a specialized high-tilt sample holder is used, projection images can only be recorded from −70° to +70° and projection information of a 40° tilt range are not accessible^1^. The limited tilt range in electron tomography (ET) is referred to as the missing wedge problem because in the 2D Fourier transform of the tomogram, there is no information transferred in a wedge-shaped area that corresponds to the missing projections in the sinogram (Fig. 1). The large missing wedge of information introduces elongation and ghost tail artifacts in the reconstructed tomograms (Fig. 1).

At the present, one of the major challenges in all tomography techniques, including electron tomography, is the incomplete or insufficient sampling in the angular/radon space, which makes the inversion problem mathematically ill-posed, i.e. there is insufficient number of linear equations to solve the linear algebraic problem, which leads to artifacts and reduction in reconstruction quality and resolution. To solve this problem, many methods have been proposed to mitigate the artifacts of inverse Radon transform or back projection. For example, the weighted back projection (WBP) method corrects back projection by applying a ramp filter that dumps the low-frequency information and enhances the high-frequency ones. WBP is an efficient and non-parameter method; however, it performs well only when there are sufficient projections available. When the angular sampling is sparse or there is a missing wedge, the WBP method introduces streaking, elongation and ghost tail artifacts. Improved upon WBP, simultaneous algebraic reconstruction technique (SART)^2–5^ is an iterative reconstruction method that can partially recover the lost projection information; however, it is ineffective at filling the lost information when a large missing wedge is present. To further mitigate the problem, algorithms that utilize borrowed ideas from the field of compressed sensing has been developed. For example, Total Variation Minimization (TVM)^6^ is one of those methods that deploys the sparsity constraint in the gradient domain of the tomograms. It combines iterative reconstruction and regularizations on Total Variance to recover the missing wedge of information and reduce streaking and ghost tail artifacts. However, the TVM method promotes piece-wise constant in the tomogram domain which makes the reconstruction patchy-looking and lacks fine and continuous tonal changes and details. To improve TVM, higher-order generalized TVs have been proposed but problems still remain—all TV-related methods are based on imposing prior constraints that may perform well on scenarios that closely satisfies the constraints and fail on others. In many cases, the TV regularization works against the projected data. A fine balance between the TV regularization and imposing the projection requirement needs to be manually found case by case. So far, the reconstruction quality of TV-based methods heavily relies on manual parameter tuning, which is often done by human operators through visual inspection. These methods can be disadvantageous for images that have complicated details where even human operators are incapable of judging the reconstruction quality. Therefore, it is hugely beneficial to design an end-to-end method that can recover the unacquired/lost information under the missing wedge conditions without any human supervision. Herein, based on generative models in deep learning, we present a joint model for the inpainting of the missing-wedge sinogram and the de-artifact of the reconstructed tomogram.

In recent years, with the explosive development of deep neural networks, many creative algorithms based on deep learning have been developed in the computer vision field, such as image transformation, object detection, segmentation, edge detection, image restoration and sharpening^7–9^, based techniques like normalization^10–13^, super-resolution^14–19^. In particular, the Generative Adversarial Networks (GAN)^20^ has been proven to be highly effective in a wide range of applications in high-dimensional data processing. Different from the traditional neural networks, a GAN network consists of two neural networks, the generative model, and the discriminative model. The generative model yields fake data through the learning of the training data and aims to fool the discriminator. The discriminator determines whether an image is a real image. The goal of the discriminator is to distinguish the “fake” images generated by the generative model from the “real” images in the training set. In our context, the real data is the sinogram without any missing wedges, denoted as the complete sinogram from here on, and the fake data is the sinograms with the missing wedge inpainted; the discriminative model will distinguish whether a sinogram is a complete sinogram or an inpainted sinogram generated by the generative model. During training, the two models contest with each other and are improved at the same time. It is worth noting that because of the discrimination process, it is possible to generate “real” data without a large amount of prior knowledge of the real data’s information distribution. As a result, the generated sinogram will eventually be too authentic to be distinguished accurately by discriminator and reach a Nash equilibrium.

**Figure 2:**
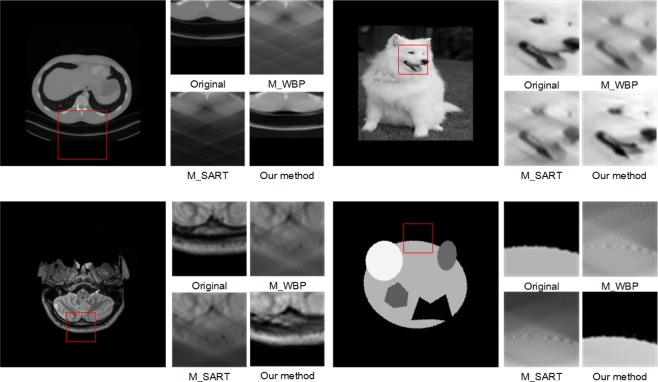
The details of Original images, missing wedge reconstruction in WBP, SART, and our method.

Compared with other methods, GAN produces more realistic images with more details and higher image quality, especially in inpainting applications. In this paper, we introduce a two-step deep GAN model to tackle the missing-wedge problem. We first design a sinogram filling model based on the use of a super-resolution reconstruction GAN^14,16–18^. Then, we use a U-net structured GAN to further reduce the residual artifacts, i.e. streaks and ghost tails, in the reconstructed tomogram. The rationale for building a two-step model is to perform information inpainting in the sinogram domain and artifacts removal in the tomogram domain, each in their respective suitable domain. The results show that our two-step GAN model can achieve outstanding Peak Signal to Noise Ratio (PSNR) and the Structural Similarity Index (SSIM) and remarkable missing wedge filling effect. As shown in Fig. 2, the two traditional methods, SART and WBP, show significant streaking and ghost tail artifacts. Nevertheless, our joint model can fill the missing information and reduce the artifacts significantly. More importantly, our method can recover the sharp boundaries where the traditional methods fail to reconstruct.

## Method

In this section, we present the construction of the two-step joint model that can efficiently recover the missing-wedge of information without introducing visible artifacts in the reconstructed sinogram. We firstly present a sinogram filling network based on Residual in Residual Dense Block (RRDB)^18^. Then, we use U-net as a de-artifact model for the removal of image artifacts after reconstruction. Both models are used in the framework of GAN, and the relativistic discriminator loss (RaGAN) is used^21^. Finally, we evaluated the PSNR and SSIM^22^ of the reconstruction results provide a quantitative benchmark of our and the reference methods.

**Figure 3:**
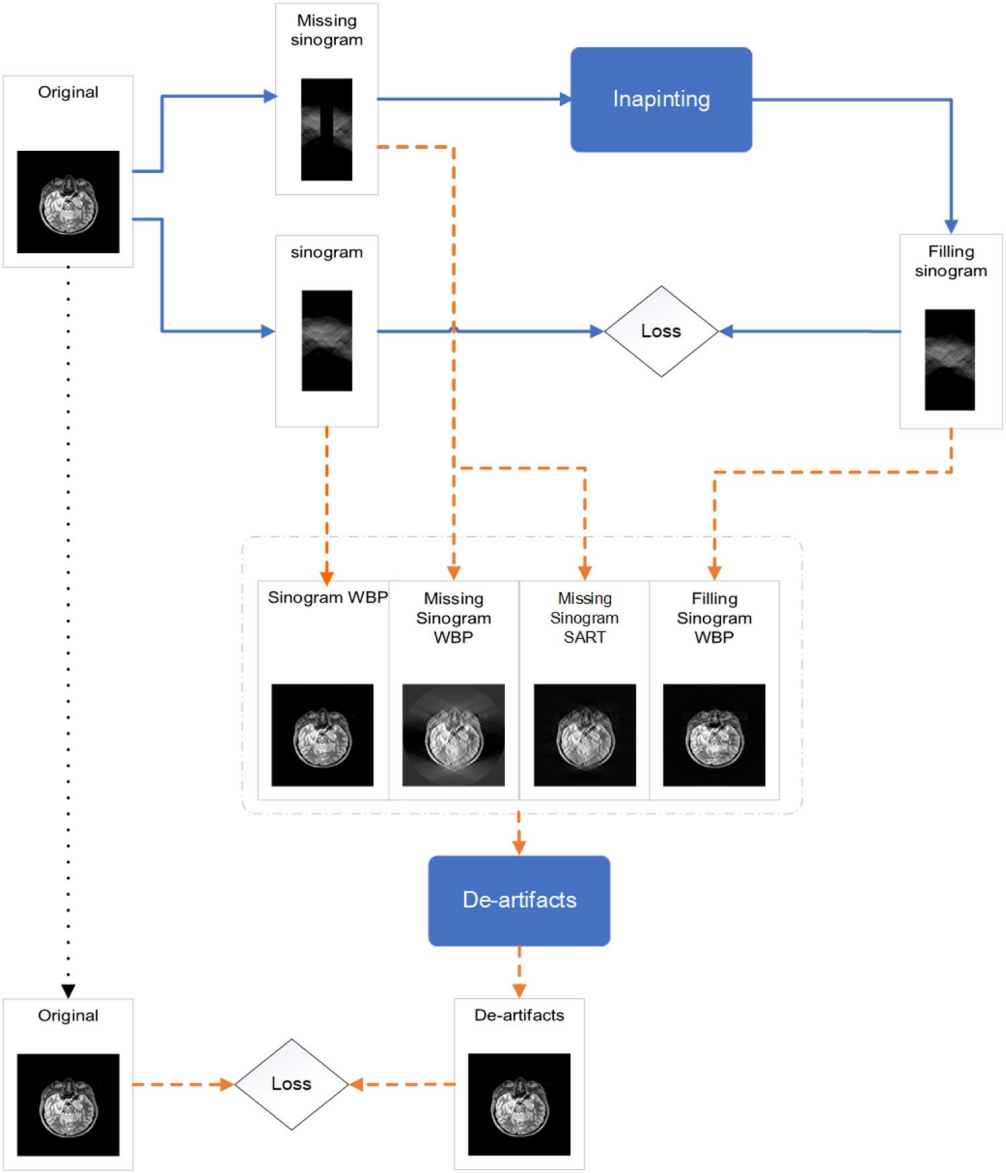
Schematics of the entire working pipeline of the joint model proposed in this article.

The working pipeline of the two-step model is shown in Fig. 3. For the missing wedge inpainting process, we perform Radon transform on a library of images to create sinograms with and without the missing wedge. The complete sinograms are used as the ground truth and the missing-wedge sinograms are used as the input of the inpainting model.

For the training of the de-artifacts model, we collect reconstructed tomograms of the missing-wedge sinograms, the ground-truth sinograms, and the inpainted sinograms. We use them as the input of de-artifacts model and the original cross-sectional images as the ground truth to compute the loss.

### Inpainting model

**Figure 4:**
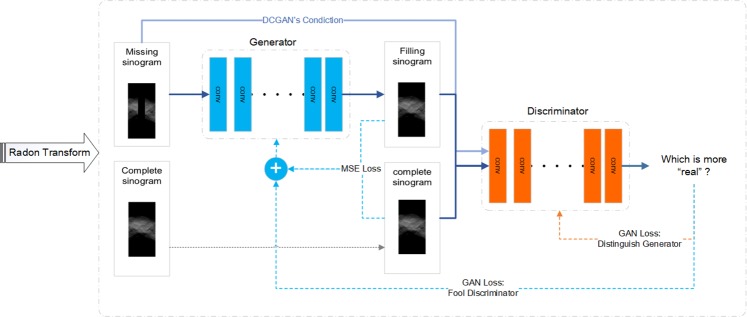
The structure of the sinogram inpainting network.

Figure 4 shows the structure of inpainting GAN model. During training, the generator learns to generate inpainted sinograms that more and more resembles the ground-truth sinograms. We compute a part of the joint loss, mean square error (MSE), by using ground-truth sinograms and inpainted sinograms. The other part of the joint loss is GAN loss. For this GAN loss, there is another technique in used, which is called CGAN^14^. This means that the inputs of discriminative model not only include the real and fake data but also include the input of the generative model.

### Data

To create a training library for the inpainting model, we first create a library of cross-sectional images. The library comprises of simulated images and images acquired from open datasets including ImageNet^23^, MGH^24^ and the National Biomedical Imaging Archive (NBIA)^25^. The simulated images are composed of random overlaps of round objects and polygons that resemble the cross-sectional images of faceted or rounded nanocrystals. In ImageNet dataset we only use images that are tagged with dogs. MGH is a public database of medical brain CT image and NBIA is a dataset of medical CT images of tumors. We use the ImageNet and brain/tumor images to increase the robustness of the network for complex and more realistic textures. The total number of the training set is 55,000. We randomly split it to 50,000 and 5,000 as training dataset and validation dataset respectively. The training samples were augmented according to the methods described in Table 1 before they were Radon transformed into sinograms. (For details on image augmentation, see Supplementary Materials).

**Table 1:** Image augmentation.

dataset\processing	Pad Resize	Radom Rotation	Radom Flip	Radom Affine	Random Noise	Size
ImageNet	√				√	10,000
Random shape	√	√			√	15,000
MGH	√	√	√	√	√	15,000
NBIA	√	√	√	√	√	15,000

**Figure 5:**
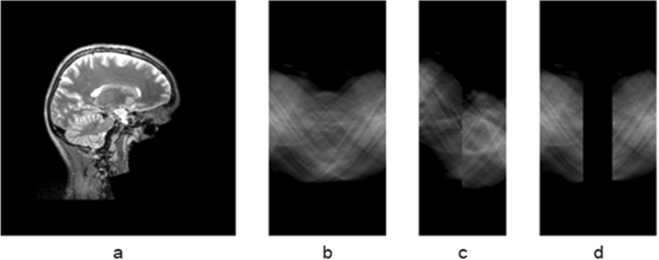
The ground-truth sinogram and missing-wedge sinogram. (**a**) The cross-sectional brain image, (**b**) complete sinogram, (**c**) missing-wedge sinogram, (**d**) missing-wedge sinogram with the missing projections padded with zeros.

Sinograms with and without the missing wedge were created by Radon transforming the library of cross-sectional images (see Supplementary Materials for detail). Figure 5 shows examples of a brain CT image from the library (Fig. 5a), the ground-truth sinogram (Fig. 5b), and sinogram missing 45 degrees of projections Fig. 5c,d).

### Network

**Figure 6:**
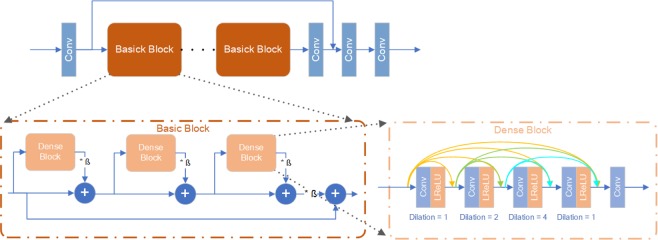
Inpainting model generator structure.

The generative model of the inpainting GAN is showed in Fig. 6. The generative network structure is mainly based on the RRDB model proposed by Xintao Wang^18^ but without the final upsampling layer. The RRDB model combines the Resnet and Densenet without applying Batch Normalization (BN) to avoid the noise from BN. In addition, it does not use pooling layers and thus retains the input information at max resolution. Compared with the standard RRDB, our model uses a dilated convolution layer to widen the receptive field.

**Figure 7:**
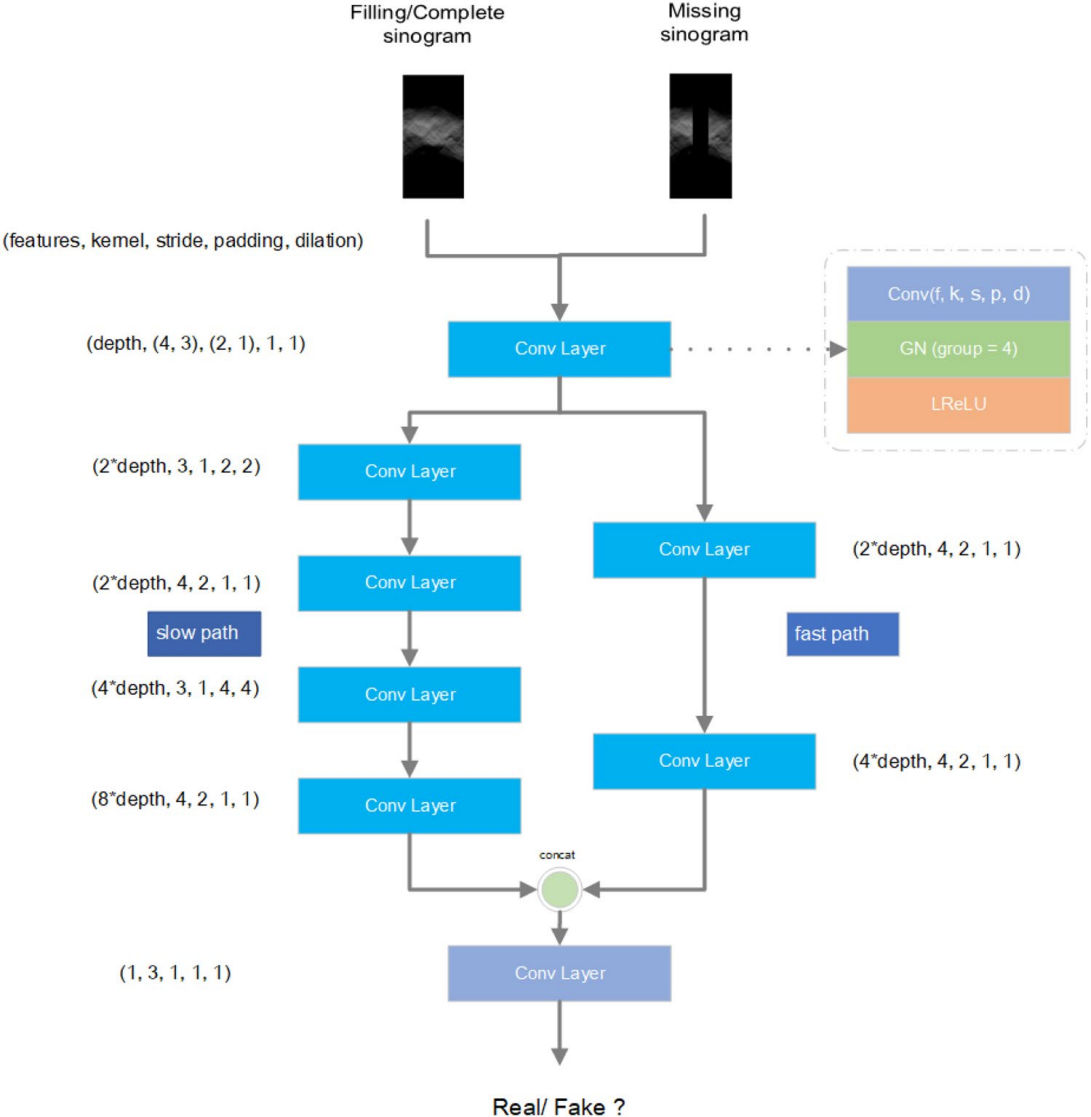
Discriminator Structure details and convolution kernel information.

Our discriminator model uses a classic convolutional layer stack, while the difference is that dual feature extraction is used-the large receptive field slow path and the small receptive field fast path. These two paths are used to extract global and local image features, and to ensure the overall and local output quality of the model. However, due to the difference in width and height of the input data, the convolution kernel of the input layer is asymmetric. At the same time, we also use dilated convolution to increase the receptive field of the model that will give more gradient information to guide the generator. As for nominalization layer, we use Group Nominalization^13^ with group 4 instead of Batch Nominalization^10^. Finally, we set feature depth as 64. The details are showed in Fig. 7.

### Loss function

We use a joint loss function consisting of MSE and GAN loss. For GAN loss, we used the least squares GAN loss^21,26^. This loss is simple and stable with lower computational cost. (See supplementary materials for details).

### Training strategy

The total training epochs are 30. During the training, we set the ratio of the discriminative model and the generative model training frequency as 1:1. For the first three epochs, we set learning rates as 1e-4, 2e-4, and 4e-4, for both the generative and the discriminative models. Then the rate decays at the 20th and 28th epochs multiplied by 0.1. The optimizers and hyper-parameters are shown in Table 2. We set minibatch size 8 and using two Nvidia 1080TI GPUs. After each epoch training, we validate the training process by validation dataset, and then evaluate the SNR and SSIM score.

**Table 2:** The training optimizer and hyper-parameters.

Model\hyper params	Optimizer	learning rate	weight decay	betas	momentum	alpha
Generator	Adam	4e-4	1e-4	(0.9, 0.999)	/	/
Discriminator	RMSprop	4e-4	1e-4	/	0	0.99

### De-artifacts model

The inpainted sinogram is expected to provide improved reconstruction quality because of the recovery of the missing wedge of information. However, when we use WBP or SART to reconstruct the tomograms, there are still residual streaking and ghost tail artifacts in the reconstruction. The residual artifacts are a result of any small deviations of the inpainted sinogram from the ground truth. So, the goal of this model is to reduce the residual artifacts in the final tomogram.

### Data

Our training dataset consists of the following four subsets. The total size is 45000. We randomly choose 5000 samples as the verification dataset. The detail is shown in Table 3.

- Tomograms reconstructed from the missing-wedge sinograms using WBP. Size is 10,000.
- Tomograms reconstructed from the complete sinograms using WBP. Size is 7,500.
- Tomograms reconstructed from the missing-wedge sinograms using SART. Size is 7,500.
- Tomograms reconstructed from the output of inpainting model using WBP. Size is 20,000.

**Table 3:** De-artifacts model training dataset.

Condition of sinogram	WBP	SART
Missing wedge	10,000	7,500
Complete	7,500	/
Inpainted	20000	/

**Figure 8:**
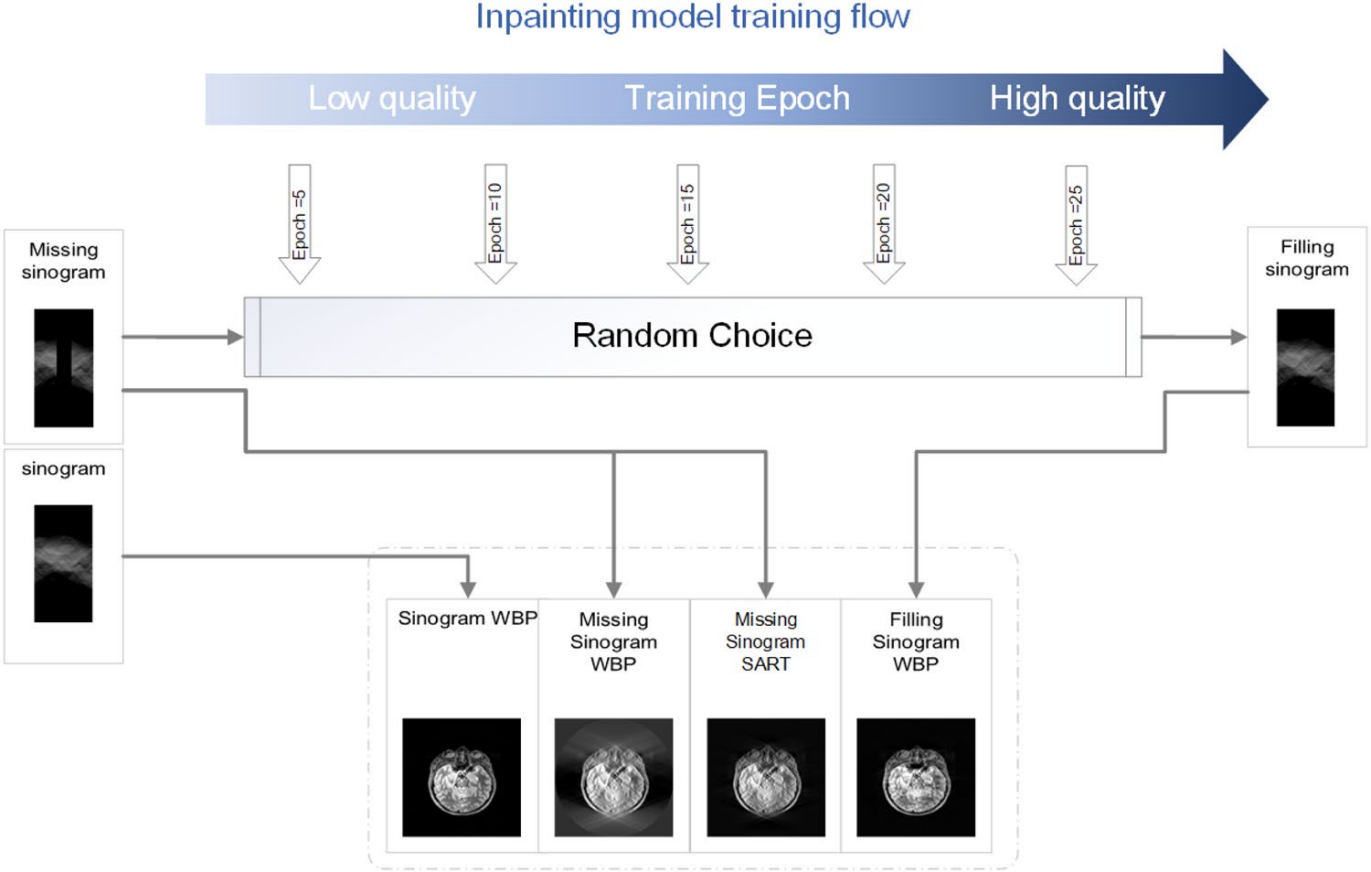
The training data of the denoising model.

The primary purpose of the de-artifacts model is to remove the artifacts yielding from the reconstruction process after filling the sinogram. So, the images generated via inpainting model is the core of the training data set. The inpainting models at different checkpoints are used to generate inpainted sinograms, followed by WBP reconstruction as shown in Fig. 8. By choosing a few different checkpoints of the inpainting model, we can obtain multiple different levels of inpainting effect (The later the checkpoint is, the stronger the inpainting effect is) of sinogram to improve the robustness of the de-artifact model.

A small number of missing-wedge sinograms are directly transformed by WBP and SART. It will generate slightly different artifact patterns to improve the robustness on the de-artifact model. We also included tomograms reconstructed by WBP from the complete sinograms. There are fewer artifacts in these tomograms. By using these images in the training dataset, over-de-artifacting can be prevented. In other words, the false positive rate is reduced. It will also prevent the model from overfitting.

### Network

The generation model in the denoising GAN is a standard U-net structure^27^. Its encoding followed with decoding mode can effectively remove artifacts and noise in the original image. At the same time, the cross-layer connection can speed up the flowing of feature information and reduce the loss of feature information. The structure is shown in Supplementary Fig. S1.

As for discriminator, it has a similar structure with the discriminator in inpainting GAN, as shown in Supplementary Fig. S2. This a standard convolution layer stack. We keep using dilated convolution and set dilation equal to two. Before the output layer, the last convolution layer uses Max pooling rather than Average pooling.

However, this time we replace Group Normalization with Batch Normalization. Because this model requires less memory. So, we can set much larger minibatch size to attenuate the noise yield from BN. The training details are in Supplementary Table S1.

## Result

**Figure 9:**
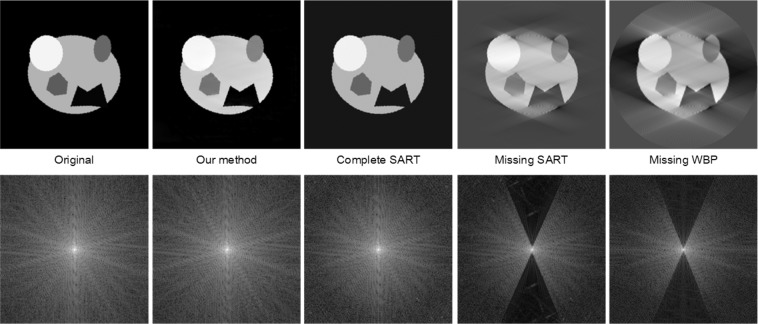
The comparing of reconstruction images and fast Fourier transformed (FFT) images. From left to right are the original image, Inpainting-de-artifacted (our method) image, complete sinogram with SART, missing-wedge sinogram with SART, missing-wedge sinogram with WBP.

Figure 9 shows the tomograms reconstructed by our joint model and other benchmarking methods. The result shows that our method readily fills the missing wedge of information and near perfectly reconstruct the image of random geometrical shapes. On the other hand, the missing wedge leads to prominent artifacts in the tomograms reconstructed by SART or WBP methods. It is worth noting that our method is capable of filling the missing wedge information up to the high spatial frequencies, which is partly lost in the SART reconstruction (Fig. 9).

**Figure 10:**
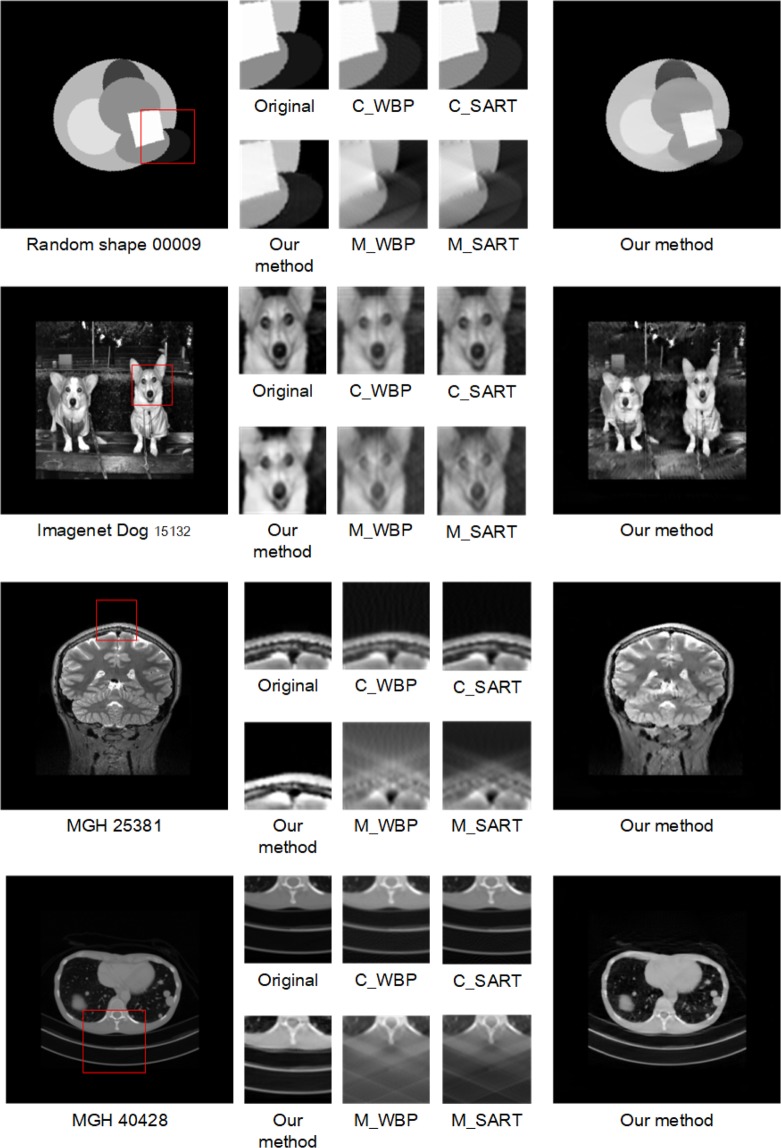
The comparing of reconstruction effect of complete sinogram (C_), missing-wedge sinogram (M_) in WBP, SART and our method.

Despite the outstanding performance of our method in reconstructing random geometrical shapes, some of the experimental images can be far more complex and details-abundant, and therefore to reconstruct these images requires the reconstruction algorithms to self-adapt to the sceneries such requirements renders conventional methods or even some of the state-of-the-art TVM methods ineffective. Figure 10 shows the comparison of reconstruction tomograms of such complex scenarios from ImageNet and MGH by our joint model and the benchmark methods. It is visually obvious that our method provides superior reconstruction results. The outstanding results suggest our model is highly robust and can self-adapt to different scenarios without having to choose hyperparameters which is a known to be the strength of deep GAN models.

To systematically evaluate the performance of our method compared with other reconstruction approaches, we investigate the Peak Signal to Noise Ratio (PSNR), the Structural Similarity Index (SSIM), and Perceptual Index (PI)^28^ of the tomograms reconstructed by our joint model, WBP, SART, and TVM from the complete and the inpainted sinograms, respectively.

**Figure 11:**
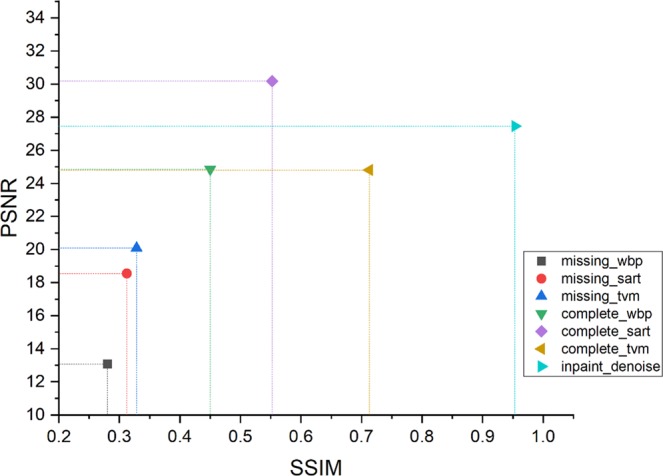
Plot of SSIM and PSNR. Upper right is better than lower left.

Figure 11 is the PSNR vs. SSIM plot of the different methods^22^. The detail is shown in Table 4.Our method shows the best performance among all the methods for reconstruction from the missing-wedge sinograms. Most amazingly, it even outperforms images reconstructed from complete sinograms via WBP and TVM.

**Table 4:** Peak Signal to Noise Ratio (PSNR) and Structural Similarity Index (SSIM) of the joint model and the benchmarking methods plotted in Fig. 11.

Method	PSNR	SSIM
missing_wbp	13.07	0.2804
missing_sart	18.55	0.3124
missing_tvm	20.09	0.3283
complete_wbp	24.84	0.4499
complete_sart	**30.17**	0.5522
complete_tvm	24.81	0.7130
**this method**	**27.46**	**0.9532**

We also benchmarked out method using the Perceptual Index (PI) and Root Mean Square Error (RMSE). The perceptual quality is judged by the non-reference measurements of Ma’s score^29^ and Naturalness Image Quality Evaluator (NIQE)^30^. This unified approach quantifies the accuracy and perceptual quality of algorithms jointly^28^.

$${\rm{perceptual}}\,{\rm{index}}\,({\rm{PI}})=1/2\,((10-{\rm{Ma}})+{\rm{NIQE}})$$

**Figure 12:**
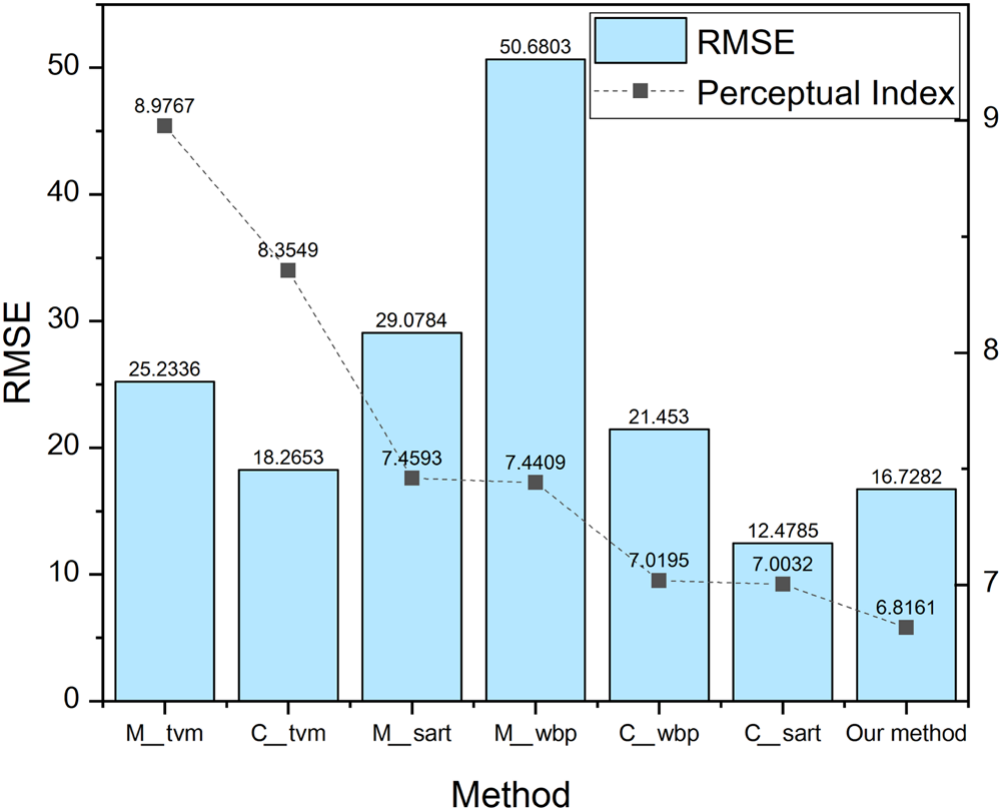
Perceptual Index and RMSE of tomograms reconstructed by our joint model and the benchmarking methods from the missing-wedge sinograms (M_) and complete sinograms (C_).

In Fig. 12, our method has the lowest PI compared to that of other reconstruction methods in all conditions, and the quantitative numbers are listed in Table 5. It means that our method has the best perceptual quality in the reconstruction of missing-wedge sinograms, which is even better than the quality of SART reconstruction of the complete sinograms because both SART and WBP involve algebraic operation, leading to the artifacts that cannot be removed by themselves even with complete sinograms. However, our approach can easily eliminate the artifacts and achieve better perceptual image quality. As for RMSE, which represents the quantitative deviation of the reconstructed tomograms from the ground truth images, our method also shows outstanding performance, and the RMSE of our jointed model is only slightly higher than that of the SART reconstruction of the complete sinograms. In conclusion, by using quantitative measurements (e.g. PSNR/SSIM) and Perceptual Index, we show that that our joint model presents the highest perceptual reconstruction quality and a markable objective quality score among all the benchmarking reconstruction methods.

**Table 5:** Perceptual Index and RMSE of the joint model and the benchmarking methods plotted in Fig. 12.

Method	Perceptual Index	RMSE
missing_tvm (M_tvm)	8.9767	25.2336
complete_tvm (C_tvm)	8.3549	18.2653
missing_sart (M_sart)	7.4593	29.0784
missing_wbp (M_wbp)	7.4409	50.6803
complete_wbp (C_wbp)	7.0195	21.453
complete_sart (C_sart)	7.0032	**12.4785**
**Our method**	**6.8161**	**16.7282**

**Figure 13:**
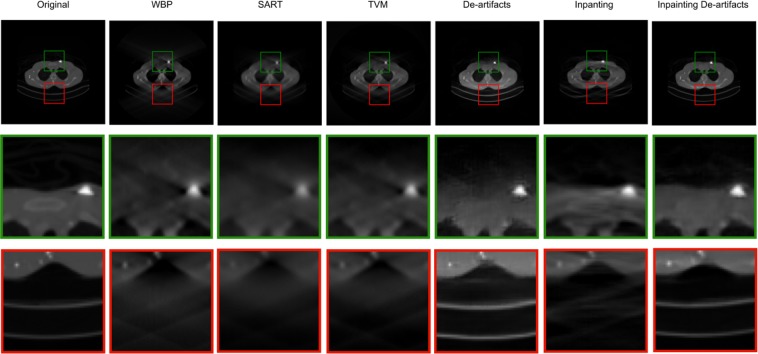
Comparison of the reconstruction results of the de-artifacts, inpainting, and joint model.

Further, we explore how these two models, the inpainting network and the de-artifacts network, work separately and jointly. We find that the inpainting process make the de-artifacts process more robust and easier to recover the details. As shown in Fig. 13, using only the de-artifacts model leads to blurred boundaries (show in the green boxes) and a poor intensity recovery (shown in the red boxes). For sinogram inpainting model alone, the reconstruction still has residual artifacts because the information filling is done in the sinogram space where the weighting of the errors is different from that of the tomogram space. But if two models work jointly, the inpainting output can make the de-artifacts process more robust both in terms of edge recovery and intensity accuracy (Fig. 13).

**Figure 14:**
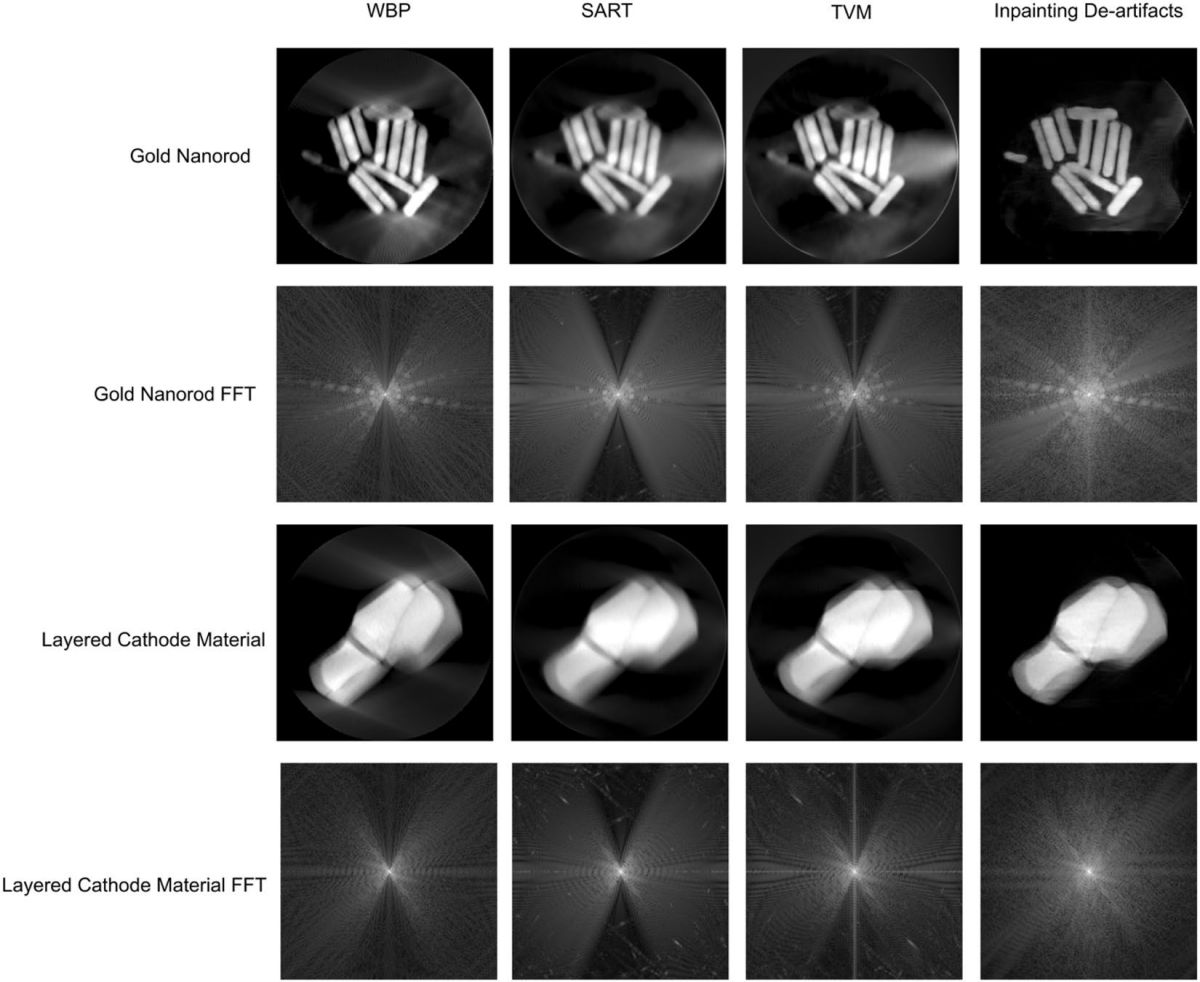
Tomograms of gold nanorod and layered cathode material reconstructed by WBP, SART, TVM and the joint model.

Finally, our model is built based on simulated data. So, we tested our method using experimental data of gold nanorods and layered cathode materials. The results are shown in Fig. 14, even though these data have never been used in training, our joint model clearly outperforms other methods.

## Conclusion

The reconstruction artifacts of limited-tilt range tomography are largely due to loss of information in the missing wedge. The lost of information is also manifested in the sinogram—a range of projection information is unavailable making the tomography inverse problem ill-posed. In this paper, we show that the unacquried projection information can be effectively recovered in the sinogram domain using an inpainting GAN model through learning from thousands of sinograms. However, the imperfection of the inpainted information can still lead to artifacts. To fully resolve the problem, we designed a second GAN network that removes residual artifacts in the tomogram domain. By combining the two networks into a joint model, it achieves remarkable tomography reconstruction quality for missing-wedge sinograms with a missing angle as large as 45 degrees. The improved performance of our model stems from the fact that we decouple the problem into two separate domains. In each domain, a unique solution can be learned efficiently. In addition, our method is parameter free. Its performance is independent of parameters turning, prior knowledge, or the human operator’s experience.

## Supplementary information

Supplementary info

## Data Availability

The datasets generated and analyzed during the current study are available from the corresponding author on reasonable request.

## Code Availability

The code generated during the current study are available from the corresponding author on reasonable request.
