# Supplementary material for: A joint deep learning model to recover information and reduce artifacts in missing-wedge sinograms for electron tomography and beyond
Source: Sci Rep. 2019 Sep 5;9:12803. doi: 10.1038/s41598-019-49267-x (PMC6728317; doi:10.1038/s41598-019-49267-x)
Supplement: Supplementary file 1 — Supplementary info [file 41598_2019_49267_MOESM1_ESM.pdf]

Online supplementary material for

A joint deep learning model to recover information and reduce artifacts in missing-wedge sinograms for electron tomography and beyond

Guanglei Ding[1,2,‖], Yitong Liu[2,‖], Rui Zhang[1], Huolin Xin[1]
1. Department of Physics and Astronomy, University of California, Irvine, CA 92697, United States
2. School of Information and Communication Engineering, Beijing University of Posts and Telecommunications, Beijing 100876, China
‖ These authors contributed equally
Correspondence should be addressed to HLX (huolin.xin@uci.edu)

This document contains:
Supplementary methods and supplementary figures

Supplementary methods:

Detains on the augmentation of the training images

- Pad Resize. The purpose of Resize and Pad is to ensure that the original image is within the range of the rotated projection during the radon transformation. The output shape is (1, 256, 256).
- Random Rotation. Random rotate the image in 0 to 180 degree.
- Random Flip: Random flip in vertical and horizontal with probability of 50%.
- Random Affine: Random affine, which is used in "torchvision.transforms. RandomAffine ". The parameters are:
  - degrees = 90,
  - translate = (0, 0.05),
  - scale = (0.85, 1.15),
  - share = 10
    - Random Noise: We add noise after Radon transform of input training images.
      - 50% data: No noise.
      - 20% data: Add Gaussian Noise (mean = 0, std = 0.002)
      - 20% data: Add Poisson Noise
      - 10% data: Add both.

Details on the sinogram preparation

Radon transform: We set degree step as 1.40625° from 0° to 180° with 128 projections. So, the complete shape of sinogram is (1, 256, 128) that will be used as the ground truth during the training. The missing wedge sinograms are built from 67.5° to 180° concatenated with 112.5° to 180° at the same delta. The shape is (1, 256, 96) which means that we lose the middle 45 degrees wedge and 32 projections. However, to align the image dimension of the input and output, we use a constant to fill the missing part of the center data, and the value of this constant is the mean value of all pixels of the narrow sinogram.

Details on the generative model of the in-painting network

- Expand the receptive field. We use dilated convolution and modify the dilation of each layer to expand the receptive field. Lacking the receptive field will result in the inability to reconstruct the information accurately[1].
- Remove the pixel shuffle layer. The end of the pixel shuffle layer is removed because we need to fill the gaps with information, so there is no need for an up-sample layer.

For the model parameters, all the layers kernels size is (3, 3), stride equal to 1, and we set padding equal to 1 to keep the same dimension. We use 16 Basic Block in this model, and basic number channels and the growth rate in Dense block are 16.

Details on the loss function of the in-painting network

x_{input} represents the input data. The generator and the discriminator are denoted as G and D. x_{fake} is the output of generator. We use x_{real} to represent the ground truth.

The loss of standard GAN is used to distinguish if the sinogram is real or fake. Here, we replace the standard discriminator loss with the Relativistic GAN loss. The relativistic discriminator tries to predict the probability that a real image is relatively more realistic than a fake one. The relativistic discriminator will compensate for the missing information from standard GAN.

Standard GAN loss for D:

$$L_D(x_{real}) = D(x_{real}) \rightarrow 1 \Rightarrow Real \quad (1)$$

$$L_D(x_{fake}) = D(x_{fake}) \rightarrow -1 \Rightarrow Fake \quad (2)$$

Based on this, we get the Least Square Loss function:

$$min\ L_D = [D(x_{real}) - 1]^2 + [D(x_{fake}) + 1]^2 \quad (3)$$

RaGAN loss for D:

$$L_{RaD}(x_{real}) = D(x_{real}) - E[D(x_{fake})] \rightarrow 1 \Rightarrow More\ Real \quad (4)$$

$$L_{RaD}(x_{fake}) = D(x_{fake}) - E[D(x_{real})] \rightarrow 0 \Rightarrow Mor\ Fake \quad (5)$$

Then we apply relativistic loss with LSGAN[2] (RaLSGAN[3]) combined with the idea of RaGAN proposed by Alexia Jolicoeur-Martineau[3]. Eventually, we define the discriminator loss as:

$$min\ L_{RaD} = [D(x_{real}) - E[D(x_{fake})] - 1]^2 + [D(x_{fake}) - E[D(x_{real})] + 1]^2 \quad (6)$$

The E [·] represents the average operation of minibatch when loading from the dataset.

The adversarial loss for the generator is in a symmetrical form:

$$min\ L_{RaG} = [D(x_{real}) - E[D(x_{fake})] + 1]^2 + [D(x_{fake}) - E[D(x_{real})] - 1]^2 \quad (7)$$

Besides, we can get MSE loss as:

$$L_{MSE} = (x_{fake} - x_{real})^2 \quad (8)$$

So, the final loss function for the generator is:

$$min\ L_G = L_{RaG} + ß\ L_{MSE} \quad (9)$$

$$ß = 0.004$$

MSE is used directly to generated fake images and real images, and RaLSGAN is used as the GAN loss. Since the ß is small, the same value in [1], the MSE weight is large, therefore the reduction of the loss function in the initial stage of training mainly depends on the reduction of the MSE which will help stabilize the GAN's training. When the MSE is decreased to a certain extent, the GAN loss will play a role and gradually improve the sensory quality of the image.

Details on the De-artifacts network

Loss function

The loss function is the same with inpainting training loss while without the MSE part.

$$min\ L_{RaD} = \left[D(x_{real}) - E\left[D\left(x_{fake}\right)\right] - 1\right]^2 + \left[D\left(x_{fake}\right) - E[D(x_{real})] + 1\right]^2 \quad (10)$$

$$min\ L_{RaG} = \left[D(x_{real}) - E\left[D\left(x_{fake}\right)\right] + 1\right]^2 + \left[D\left(x_{fake}\right) - E[D(x_{real})] - 1\right]^2 \quad (11)$$

Training strategy

Theoretically, the joint model should be training together. Because the training of de-artifacts model relies on the output data of inpainting model. However, we don't have enough memory to load these models simultaneously. We train the inpainting model first and then we save the output data and model checkpoint at different epoch. So, we can train these two parts models separately.

Most of the training strategies are the same as previous works. The total training epochs are 30. For the first three epochs, we set learning rate 1e-4, 1.5e-4, and 2e-4, for both generator and discriminator. Then we keep 2e-4 as a constant learning rate, and we still verified each epoch by computing SNR and SSIM score.

We set minibatch size 32 and using two 1080TI GPUs.

Table S1: The optimizer and hyper-parameters in denoise training.

Model\hyper params	Optimizer	learning rate	weight decay	betas	momentum	alpha
Generator	Adam	2e-4	5e-4	(0.9, 0.999)	/	/
Discriminator	RMSprop	2e-4	5e-4	/	0	0.99

Supplementary figures:

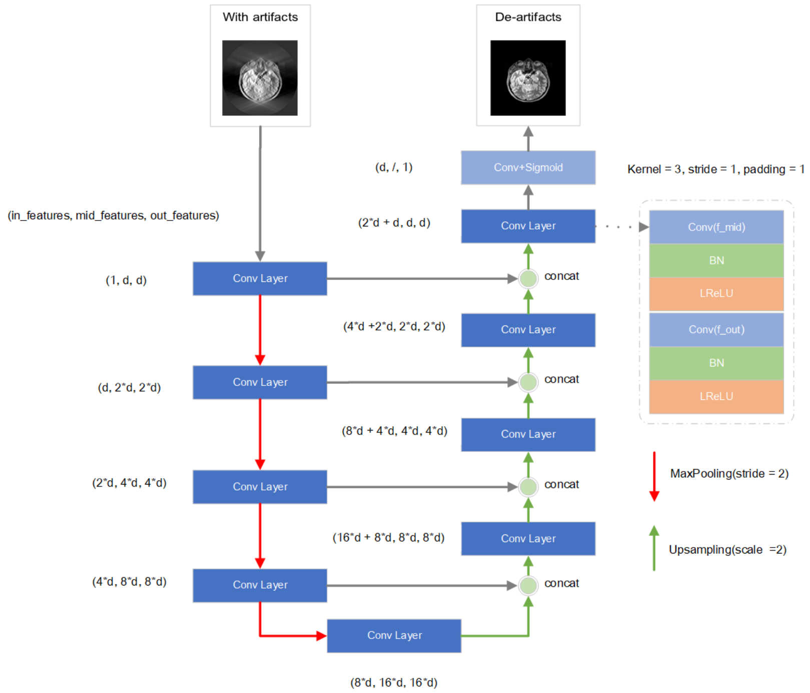

Figure S1. The standard u-net structure for de-artifacts generator.

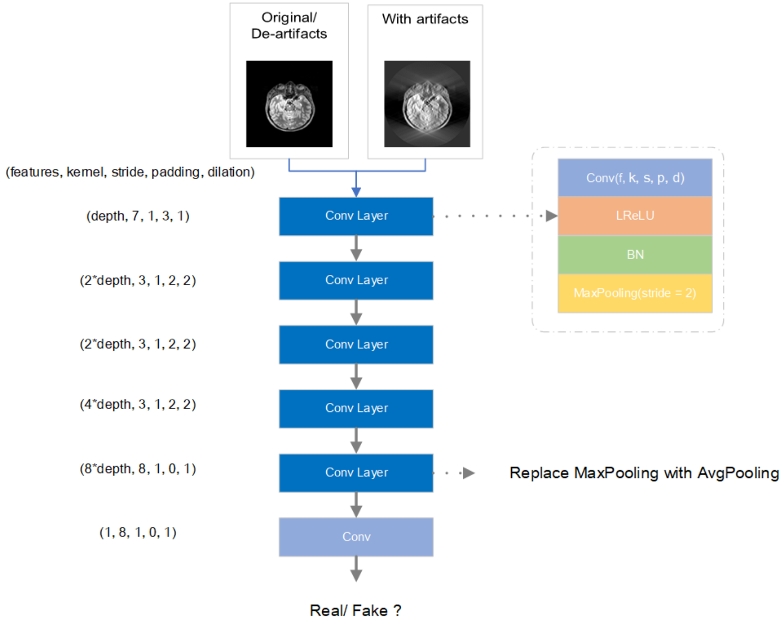

Figure S2. The structure of discriminator.

Supplementary references:

1 Sabini, M. & Rusak, G. Painting Outside the Box: Image Outpainting with GANs. *arXiv preprint arXiv:1808.08483* (2018).

2 Mao, X. *et al.* in *Proceedings of the IEEE International Conference on Computer Vision.* 2794-2802.

3 Jolicoeur-Martineau, A. The relativistic discriminator: a key element missing from standard GAN. *arXiv preprint arXiv:1807.00734* (2018).
